# Synergistic Combination of *Citrus* Flavanones as Strong Antioxidant and COX-Inhibitor Agent

**DOI:** 10.3390/antiox12040972

**Published:** 2023-04-21

**Authors:** Antonella Smeriglio, Nunzio Iraci, Marcella Denaro, Giuseppina Mandalari, Salvatore Vincenzo Giofrè, Domenico Trombetta

**Affiliations:** Department of Chemical, Biological, Pharmaceutical and Environmental Sciences, University of Messina, Viale Ferdinando Stagno d’Alcontres 31, 98166 Messina, Italy; marcella.denaro@unime.it (M.D.); giuseppina.mandalari@unime.it (G.M.); salvatorevincenzo.giofre@unime.it (S.V.G.); domenico.trombetta@unime.it (D.T.)

**Keywords:** *Citrus* flavanones, antioxidant activity, anti-inflammatory activity, ROS, TBARS, GSH/GSSG, carbonylated proteins, COX, molecular modeling studies, Caco-2 cell

## Abstract

Recently, we demonstrated that a *Citrus* flavanone mix (FM) shows antioxidant and anti-inflammatory activity, even after gastro-duodenal digestion (DFM). The aim of this study was to investigate the possible involvement of the cyclooxygenases (COXs) in the anti-inflammatory activity previously detected, using a human COX inhibitor screening assay, molecular modeling studies, and PGE2 release by Caco-2 cells stimulated with IL-1β and arachidonic acid. Furthermore, the ability to counteract pro-oxidative processes induced by IL-1β was evaluated by measuring four oxidative stress markers, namely, carbonylated proteins, thiobarbituric acid-reactive substances, reactive oxygen species, and reduced glutathione/oxidized glutathione ratio in Caco-2 cells. All flavonoids showed a strong inhibitory activity on COXs, confirmed by molecular modeling studies, with DFM, which showed the best and most synergistic activity on COX-2 (82.45% vs. 87.93% of nimesulide). These results were also corroborated by the cell-based assays. Indeed, DFM proves to be the most powerful anti-inflammatory and antioxidant agent reducing, synergistically and in a statistically significant manner (*p* < 0.05), PGE2 release than the oxidative stress markers, also with respect to the nimesulide and trolox used as reference compounds. This leads to the hypothesis that FM could be an excellent antioxidant and COX inhibitor candidate to counteract intestinal inflammation.

## 1. Introduction

Flavanones represent the most widespread subclass of flavonoids in the *Citrus* genus, and their antioxidant and anti-inflammatory properties are undoubted and universally recognized [1,2,3,4]. These promising activities make flavanones important candidates in the treatment of various chronic inflammatory disorders such as cardiovascular and metabolic diseases [5,6,7,8,9,10,11]. In the last decade, flavanones have been the subject of in-depth studies for the treatment of intestinal bowel diseases (IBD) due to the increase in their incidence globally, and in relation to the emergence of new therapeutic strategies that limit the range of inter-individual variability in the therapeutic response to the incidence and severity of the side effects of conventional drugs [7,8,12,13]. Indeed, several epidemiological studies show that there is a direct correlation between a diet rich in flavanones and the decrease in IBD incidence [14]. This is probably due to their activity as inhibitors of various proteins and inflammatory pathways, their antioxidant and free-radical scavenging effects, and their ability to influence the composition of the intestinal microbiota [13]. These health properties have also been demonstrated in vivo by several studies on mouse models of IBD, where the administration of extracts and juices of *Citrus* fruits, or their most representative flavanones, confirm their ability to counteract oxidative stress and inflammation [15,16,17,18,19]. Although a rather vast body of literature on the subject is available, to date, only one study has evaluated and compared the antioxidant and anti-inflammatory effects of the most representative flavanones of the *Citrus* genus [20]. Indeed, except for naringenin and hesperidin, the other flavanones remain poorly investigated. Furthermore, the information regarding the bioaccessibility of this class of molecules remains rather lacking, and knowledge about their intestinal local anti-inflammatory action, systemic absorption and the mechanisms involved in the transport of these molecules through the gastrointestinal tract, as well as their fate following digestion, would be desirable [21].

Recently, our research group carried out an antioxidant and anti-inflammatory screening of the most representative flavanones of the *Citrus* genus, highlighting which molecules were the most promising [20]. Furthermore, the same tests were also carried out on a mix of the most powerful flavanones (FM) to evaluate the potential combination effect, which has been demonstrated experimentally [20]. FM was also subjected to in vitro simulated digestion (DFM) to evaluate the potential intestinal bioaccessibility of these molecules, demonstrating how the investigated flavanones can reach the intestinal epithelium unchanged, where they can exert strong anti-inflammatory properties, as demonstrated on a Caco-2 monolayer model, in which they significantly decreased the release of various inflammatory mediators such as interleukin (IL)-6, IL-8, and nitric oxide (NO) [20].

Considering this, the aim of the present study was to upgrade the previous one, postulating a feasible anti-inflammatory mechanism of action of these molecules and investigating the ability of these five flavanones and DFM to counteract oxidative stress in the same Caco-2 cell-based model mentioned above.

To this end, in vitro enzymatic tests were first carried out to evaluate the inhibitory activity of each flavanone (neoeriocitrin, eriocitrin, hesperetin, hesperidin, and neohesperidin) and DFM on the two human COX isoforms (COX-1 and COX-2). Molecular modeling studies were carried out to highlight any interactions at the active or other binding sites of the enzymes’ isoforms. Furthermore, the downstream activity on COX was evaluated by recording the release of PGE2 by Caco-2 cells. The ability of flavanones and DFM to counteract cellular oxidative stress was investigated by monitoring the protein carbonyl content, thiobarbituric acid-reactive substances (TBARS), reactive oxygen species (ROS), and reduced glutathione/oxidized glutathione ratio (GSH/GSSG). Finally, the potential synergistic effect of DGM on all the antioxidant and anti-inflammatory assays carried out, was evaluated.

## 2. Materials and Methods

### 2.1. Chemicals

Dulbecco’s modified eagle medium (DMEM), phosphate buffered saline (PBS) pH 7.4, and 3-(4,5-dimethylthiazol-2-yl)-2,5-diphenyltetrazolium bromide (MTT) were purchased from VWR (Milan, Italy). Nimesulide, trolox, 2′,7′-dichlorofluorescin diacetate (DCF-DA), and lucifer yellow (LY) CH dilithium salt were purchased from Merck KGaA (Darmstadt, Germany). HPLC-grade standards (purity ≥ 98%) of eriocitrin (ERI), neoeriocitrin (NER), hesperidin (HED), neohesperidin (NHE), and hesperetin (HET) were purchased from Extrasynthese (Genay, France).

### 2.2. Test Solutions Preparation

Based on the results of the preliminary study by which ERI, NER, HED, NHE, and HET were selected as the most powerful antioxidant and anti-inflammatory *Citrus* flavanones, they were also tested as FM and DFM to elucidate the potential combined biological effects, and to highlight any change after in vitro simulated human digestion [20]. FM was prepared on the assumption that the DFM, which would then be applied to in vitro cell-free and Caco-2 cell-based models to test the anti-inflammatory and antioxidant activity, had a final concentration of 10 µM, which represents the mean efficacy concentration considering the half-inhibitory concentration (IC_50_) values obtained in the preliminary experiments [20]. For this purpose, stock solutions (14 mM) of each flavanone in DMSO were mixed and diluted 10-fold with Milli-Q water to obtain the 1.4 mM FM, which was used to carried out the in vitro simulated human digestion according to Denaro et al. [20], to obtain the 10 µM DFM.

### 2.3. Inhibitory Activity on Human COX-1 and COX-2

The potential activity of the five flavanones and DFM in inhibiting COX enzymes was evaluated by means of the COX (human) Inhibitor Screening Assay Kit (Item No. 701230, Cayman Chemical Company, Ann Arbor, MI, USA). This kit directly measures prostaglandin F2α (PGF2α) by the SnCl_2_ reduction of COX-derived PGH2 produced in the COX reaction. The prostanoid product is quantified via enzyme-linked immunosorbent assay (ELISA) using a broadly specific antiserum that binds to all the major prostaglandin compounds. This assay includes human recombinant COX-1 and COX-2, allowing the user to screen specific inhibitors, eliminating the false positive leads generated by less specific methods. The assay was performed according to the manufacturer’s recommendations, using each flavanone, DFM and nimesulide, as reference selective COX-2 inhibitors, all at the same concentration (10 µM). Briefly, 10 µL of the test sample was added to COX-1 and COX-2 solutions and incubated for 10 min at 37 °C. At the end of the incubation time, 10 µL of arachidonic acid was added, triggering the hydroperoxy endoperoxide (PGG_2_) production (30 s, 37 °C). Subsequently, 30 µL of SnCl_2_ was added to stop the enzymatic catalysis and to reduce the prostaglandins produced in PGF2α. In parallel with the samples, the COX activity was evaluated under the same experimental conditions, and the values obtained were compared with the background, i.e., the same enzyme inactivated in boiling water for 3 min. The results were expressed as COX-1 and COX-2 inhibition percentage (%).

### 2.4. Molecular Modeling Studies

The protein sequences of COX-1 (P23219) and COX-2 (P35354) were download from UniProt [22] and were used to model COX-1_33-583_ and COX-2_33-583_ by homology with the experimental structure of murine COX-2 in complex with an indomethacin–ethylenediamine–dansyl conjugate (PDB ID 6BL4) [23] retrieved from the Protein Data Bank [24].

The sequences were aligned to the above-mentioned murine COX-2 through ClustalW [25] with 98% identity, 95% similarity, and 93% conservation for human COX-2 and 65% identity, 80% similarity, and 78% for human COX-1. Human COX-1_33-583_ and COX-2_33-583_ were built using SWISS-MODEL [26]. The Ramachandran plot statistics for model validation are reported in Table 1.

HET, which is the aglycone of HED and NHE, was not included in these studies since we decided to carry out a comparative analysis between the couples ERI/HED and NER/NHE. NHE, HED, NER, and ERI were downloaded as 3D SDF files from PubChem [28] with CIDs 442439, 10621, 114627, and 83489, respectively.

CCDC Hermes was used to prepare the target proteins and set the GOLD [29] molecular docking simulations up. The docking space was centered on the coordinates of the native 6BL4 ligand, and all atoms included in a 15 Å radius from the centroid were included in the binding site definition. One hundred genetic algorithm runs per ligand were run, with no early termination allowed. The population and genetic operator settings were set to auto mode, with the search efficiency set to 200%. CHEMPLP was used as a scoring function and the output poses were clustered using a 1.5 Å RMSD cutoff.

Prior to the flavanone docking simulations, the docking protocol was validated by docking the native ligand of the PDB structure 6BL4 back into the protein. Regarding COX-1, an indomethacin–ethylenediamine–dansyl conjugate was docked with an RMSD value of 2.69 Å in respect to the experimental bound conformation observed in 6BL4. The biggest contribution to the high RMSD value is due to the ~180° rotation of the dansyl-fused ring system along its bond with the sulfur atom, allowed by the mutations in the solvent-exposed binding region of COX-1. Indeed, the RMSD of the indomethacin moiety, which docks into a buried region, is as low as 0.36 Å. Regarding COX-2, the RMSD values are instead low for both the whole indomethacin–ethylenediamine–dansyl conjugate and its indomethacine moiety (0.79 and 0.35 Å, respectively).

The CHEMPLP values for the best ranking docking poses were: HED/COX-1 = 72.35; NHE/COX-1 = 63.95; NER/COX-1 = 70.52, ERI/COX-1 = 72.21; HED/COX-2 = 81.63; NHE/COX-2 = 61.65; NER/COX-2 = 79.78; ERI/COX-2 = 84.59.

The best scoring docking poses were retrieved to build ligand/COX-1 and ligand/COX-2 complexes that were submitted to molecular dynamics simulations. The eight simulated environments, i.e., NHE/COX-1, HED/COX-1, NER/COX-1, ERI/COX-1, NHE/COX-2, HED/COX-2, NER/COX-2, and ERI/COX-2, were set up and run using Desmond [30], as previously reported [31].

The systems were neutralized by Na^+^ and Cl^−^ ions, which were added until a 0.15 M concentration was reached. Simulations were run using the OPLS2005 [32] force field and solvation was treated explicitly using the TIP3P water model [33].

After the systems were relaxed using a previously reported protocol [34], 48ns long simulations were carried out at 300 K in the NPT ensemble using a Nose−Hoover chain thermostat and a Martyna−Tobias−Klein barostat (1.01325 bar), applying a 1 kcal/mol harmonic constraint on the backbone heavy atoms. The time steps were set to 2 fs, 2 fs, and 6 fs for the bonded, near, and far interactions, respectively. MD trajectories were used to estimate the difference in ligand/protein interaction energies between the two COX isoforms to address the ligand selectivity. In contrast to the use of most docking scoring methods, this method accounts for protein residue flexibility and the influence of explicit solvation on ligand binding at a moderate computational cost; however, given the many approximations it uses, it is not meant to directly correspond to the inhibitory activities exerted by the ligands. On the other hand, huge interaction energy differences between protein isoforms suggest a potential ligand selectivity. The ligand/protein molecular mechanics interaction energies (E_MM_) were estimated as follows:

E_MM_ = E_MM_LIG_angle_ + E_MM_LIG_dihedral_ + E_MM_LIG_stretch_ + E_MM_LIG_vdW_ + E_MM_LIG_elec_ + E_MM_LIG-PROT_elec_ + E_MM_LIG-PROT_vdW_ + E_MM_LIG-WTR_elec_ + E_MM_LIG-WTR_vdW_ + E_MM_LIG-IONS_elec_ + E_MM_LIG-IONS_vdW_

where E_MM_LIG_angle_, E_MM_LIG_dihedral_, and E_MM_LIG_stretch_ represent the bonded molecular mechanics energies of the ligand; E_MM_LIG_vdW_ and E_MM_LIG_elec_ represent the internal van der Waals and electrostatic molecular mechanics energies of the ligand; E_MM_LIG-PROT_elec_, E_MM_LIG-WTR_elec_, and E_MM_LIG-IONS_elec_ represent the electrostatic interaction energies between the ligand and protein, ligand and solvent, and ligand and ions, respectively; and E_MM_LIG-PROT_vdW_, E_MM_LIG-WTR_vdW_, and E_MM_LIG-IONS_vdW_ represent the van der Waals interaction energies between the ligand and protein, ligand and solvent, and ligand and ions, respectively. All energy terms are averaged over the MD simulation time.

The E_MM_ values of each simulated complex were finally used to calculate the interaction energy differences for each ligand between COX-2 and COX-1, as follows:

ΔE_COX-2-COX-1_ = E_MM_(COX-2) − E_MM_(COX-1) (1)

### 2.5. Cell-Based Assays

#### 2.5.1. Cell Model

Experiments were carried out on Caco-2 cell monolayers (CacoReady™, Readycell, Barcelona, Spain) according to Denaro et al. [35]. Briefly, 8.5 × 10^4^ cells/cm^2^ Caco-2 cells (passage number 45–55) were seeded on polyester permeable supports (6.5 mm, 0.33 cm^2^, and 0.4 µm) in 24-well HTS plates (Corning Incorporated, Corning, NY, USA). DMEM low glucose (1 g/L) with 10% fetal calf serum (FCS), 2 mM glutamine, 1 U/mL penicillin, and 1 U/mL streptomycin was added onto the apical (0.3 mL) and basolateral side (0.9 mL) of each transwell support. After 21 days of culture incubation at 37 °C, 5% CO_2_, and 95% relative humidity, the Caco-2 cells were completely differentiated and polarized, resembling the morphological and functional features of mature enterocytes lining the small intestine. Before conducting the experiments, the monolayer integrity was checked by measuring the trans-epithelial electrical resistance (TEER) with a Millicell^®^ ERS-2 V/ohmmeter (Merck Millipore, Darmstadt, Germany) equipped with an STX 100C electrode (World Precision Instruments, Sarasota, FL, USA). Only Caco-2 monolayers with epithelial resistance ≥800 Ω/cm^2^ were used to carried out the experiments.

#### 2.5.2. Cell Treatment

The antioxidant and anti-inflammatory activities of ERI, NER, HED, NHE, and HET as well as DFM were evaluated on a Caco-2 transwell model, according to Denaro et al. [20]. In both cases, 25 ng/mL IL-1β was used to trigger oxidation and inflammation events. Co-treatments with each flavanone, DFM, nimesulide, and trolox as reference standards, all at the same concentration (10 μM), were carried out on the apical side by diluting the test solutions in completed DMEM (250 µL). Completed DMEM containing 0.1% DMSO and 25 ng/mL IL-1β were used as negative (CTR−) and positive controls (CTR+), respectively. Completed DMEM (0.75 mL) was added on the basolateral side and the cells were incubated for 24 h at 37 °C, 5% CO_2_, 95% relative humidity. Cell culture media were collected and stored at −80 °C until the subsequent analyses.

#### 2.5.3. Detection of PGE_2_

The activation of the arachidonic acid cascade was evaluated through the production of PGE_2_ after the addition of the arachidonic acid. Briefly, 24 h after the treatment reported above (Section 2.5.2), the cells were washed thrice with PBS and incubated with 10 mM arachidonic acid in PBS for 10 min according to Tesoriere et al. [36]. The release of PGE_2_ into the extracellular medium was quantified (pg/mL) using the Prostaglandin E2 ELISA Kit—Monoclonal (Item No. 514010, Cayman Chemical Company, Ann Arbor, MI, USA) in accordance with the manufacturer’s protocol, by using a UV–Vis plate reader (Multiskan GO; Thermo Scientific, MA, USA) set at 405 nm.

#### 2.5.4. Detection of Oxidative Stress Markers

The determination of the oxidative stress parameters was carried out on cell lysate obtained by pipetting 300 µL of cold 0.1% Triton X-100 on monolayers according to Smeriglio et al. [37]. 

The glutathione reduced (GSH) and glutathione disulfide (GSSG) quantification was carried out by solid phase extraction (SPE) followed by HPLC-DAD analysis. Briefly, 400 μL of meta-phosphoric acid was added to 200 μL of cell lysate and incubated at RT for 15 min. The sample was then centrifuged at 10,000× *g* for 5 min at 4 °C, and the supernatant cleaned up using a BOND Elut C18 cartridge 50 mg/3 mL (Agilent Technologies, Santa Clara, CA, USA) pre-conditioned with methanol (2 mL). The elution was carried out with 0.1% TFA (1 mL). The samples were filtered by a 0.22 μm nylon syringe filter and injected into an Agilent 1100 series HPLC system (Santa Clara, CA, USA). The elution was carried out on a Prodigy™ 5 μm ODS-3 100 Å LC Column 250 × 4.6 mm (Phenomenex, Torrance, CA, USA) according to Nobili et al. [38]. The quali-quantitative analysis was carried out by comparing retention times, UV–Vis spectra (range 190–400 nm), and using the external standard calibration curves (10–10,000 ng/mL) of the commercially available reference compounds (GSH and GSSG) solubilized in mobile phase.

The detection of thiobarbituric acid-reactive substances (TBARS) was carried out using the TBARS (TCA Method) assay kit (Item No. 700870, Cayman Chemical, Ann Arbor, MI, USA) following the manufacturer’s instructions. This assay was based on the fluorimetric detection of the malondialdehyde (MDA)-thiobarbituric acid (TBA) adduct, which is formed in acidic conditions and high temperatures (90–100 °C), at an excitation wavelength (λ_ex_) of 530 nm and an emission wavelength (λ_em_) of 550 nm, by a fluorescence microplate reader (FLUOstar Omega, BMG LABTECH, Ortenberg, Germany). MDA was used as a reference standard (0.0625–5 µM).

The protein carbonyl content was quantified (nM) by the protein carbonyl colorimetric assay kit (Item No. 10005020, Cayman Chemical, Ann Arbor, MI, USA) based on the 2,4-dinitrophenylhydrazine reaction, according to the manufacturer’s instructions. The amount of protein–hydrazone produced was quantified spectrophotometrically at 370 nm by using the same instrument reported above (Section 2.5.3).

The ROS levels, expressed as percentage (%), were assessed by measuring the fluorescence resulting from the intracellular oxidation of DCF-DA (10 µM in PBS), which was added to the culture medium 30 min before ending the cell treatment [37]. The medium was then removed, and the cells washed five times with PBS (pH 6.7). The cell lysates were diluted with PBS and the fluorescence recorded by the same plate reader reported above at λ_ex_ 485 and λ_em_ 535.

All data were normalized for protein concentration detected using the protein determination (BCA) kit (Item No. 701780, Cayman Chemical, Ann Arbor, MI, USA) according to the manufacturer’s instructions.

#### 2.5.5. Post-Quality Control Assays

Post-quality control assays, such as TEER measurement, as well as the apparent permeability coefficient (Papp) and paracellular flux (Pf) of lucifer Yyellow (LY) detection, were assessed to evaluate the Caco-2 monolayer integrity [20]. Cell viability was assessed by MTT assay according to Kenzaoui et al. [39].

### 2.6. Evaluation of the Synergistic Effect by CompuSyn Software

As the DFM was prepared by combining the five flavanones (ERI, NER, HED, NHE, and HET) in equimolar ratios, the potential synergistic effect of DFM in all the antioxidant and anti-inflammatory assays carried out in the present study was investigated applying the method of the constant ratio. For this purpose, five different concentrations of DFM (5, 7.5, 10, and 20 µM) were tested, maintaining the constant ratio at 2.0. In addition, the same experiments with each flavanone (ERI, NER, HED, NHE, and HET) at the same concentrations (5, 7.5, 10, and 20 µM) used in the combination experiments were carried out as a control. The activity values obtained for all assays, expressed as percentages, and converted into the fraction of effect (Fa) according to the following equation: Fa = 100% of activity/100, were used for the calculation of the synergism using CompuSyn software Version 1.0 (ComboSyn, Inc., Paramus, NJ, USA) [40]. The data points, including treatment concentrations (µM) and Fa, were automatically processed. The results, expressed as combination index (CI), quantitatively determine whether a synergism (CI < 1), an additive effect (CI = 1), or an antagonism (CI > 1) occurs in the specific experimental conditions.

### 2.7. Statistical Analysis

Five independent experiments in triplicate (*n* = 3) were carried out for both in vitro cell-free and cell-based assays. The results were expressed as mean ± standard deviation (SD). The data were analyzed by one-way analysis of variance (ANOVA), followed by Tukey’s test and the Student–Newman–Keuls method using SigmaPlot12 (Systat Software, Inc., San Jose, CA, USA). The results were considered statistically significant at *p* ≤ 0.05.

## 3. Results

### 3.1. In Vitro Inhibitory Activity on Human COX-1 and COX-2

The enzymatic assay carried out on human COX-1 and COX-2 highlighted different behaviors between the five tested flavanones (Table 2).

Indeed, the flavanones HED, NHE, and HET showed a similar trend, being active on both COX isoforms, with an inhibition of about 50%. On the contrary, NER and ERI showed a lower inhibitory activity on COX-1 and a higher selectivity towards COX-2, with an inhibition of 78% and 56.89%, respectively. All treatments showed statistically significant results (*p* < 0.001) with respect to nimesulide, used as a reference standard, which showed a marked selectivity for COX-2. Interestingly, by analyzing the data reported in Table 2, it can be noted that DFM, while maintaining the activity on COX-1 shown by the flavanones HED, NHE, and HET, showed a greater selectivity for COX-2, and was even higher in terms of inhibition percentage with respect to NER and ERI. This demonstrates, once more, how the flavanones mixed at lower concentrations to obtain a 10 µM solution exhibit increased activity with respect to the single flavanones tested at a 10 µM concentration. Finally, it is interesting to note how the activity on COX-2 is always significantly higher (*p* < 0.05 for HED and *p* < 0.001 for all other samples) than that on COX-1, both for single flavanones and DFM.

### 3.2. Molecular Modeling Studies

The structural differences between the COX-1 and COX-2 binding sites have provided valuable guidelines for the identification of COX-2 selective inhibitors by molecular modeling studies [41]. The active sites of COX-1 and COX-2 are very similar, but COX-2 has a larger binding cavity than COX-1. This is mainly due to the presence of a second pocket within the COX binding site, which is more accessible in COX-2 because of the replacement of the Ile523 residue in COX-1 with a smaller side chain residue of valine. The substitution of this residue, besides making the binding site bigger, causes conformational changes to the Tyr355 residue, which opens an additional hydrophobic pocket consisting of Leu352, Ser353, Tyr355, Phe518, and Val523 residues [39]. Access to this additional pocket is favored by a further substitution of isoleucine with a valine at position 434, whose side chain packs against Phe518, creating a molecular gate that opens a second hydrophilic pocket [42]. In contrast, in COX-1, this gate is closed due to the bulkier side chain of isoleucine. In this way, the amino acid at position 434 contributes significantly to the selectivity. Another structural difference is recorded at position 513, where histidine is replaced by arginine in COX-2, providing a positive charge and thus changing the chemical environment of the binding site and offering a determining factor in selectivity over COX-1 [43].

To obtain insight into the interactions between cyclooxygenases and the targeted compounds and examining the difference in their selectivity toward the two COX isoforms, molecular docking and molecular dynamics (MD) studies were carried out using homology models of human COX-1 and COX-2. The two models were built by homology with the experimental crystal structure of murine COX-2 in complex with a bulky ligand (PDB ID 6BL4) [23], i.e., the indomethacin–ethylenediamine–dansyl conjugate (see Section 2.4). The choice of a murine enzyme rather than a human one was based on the size of the native ligand. 

**Figure 1:**
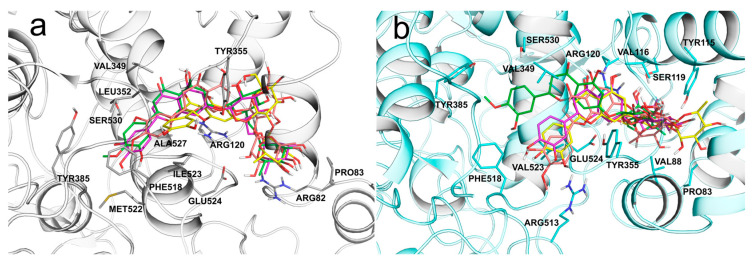
Docking poses of HED (green sticks), ERI (magenta sticks), NHE (orange sticks), and NER (yellow sticks) into (**a**) COX-1 (gray cartoons and sticks) and (**b**) COX-2 (cyan cartoons and sticks).

The four flavanones (HED, NHE, NER, and ERI) were initially studied using molecular docking simulations. The best docking poses generated by the GOLD software [29] (see Section 2.4) showed that the four phytocompounds might bind the same region in both COX isoforms (Figure 1).

**Figure 2:**
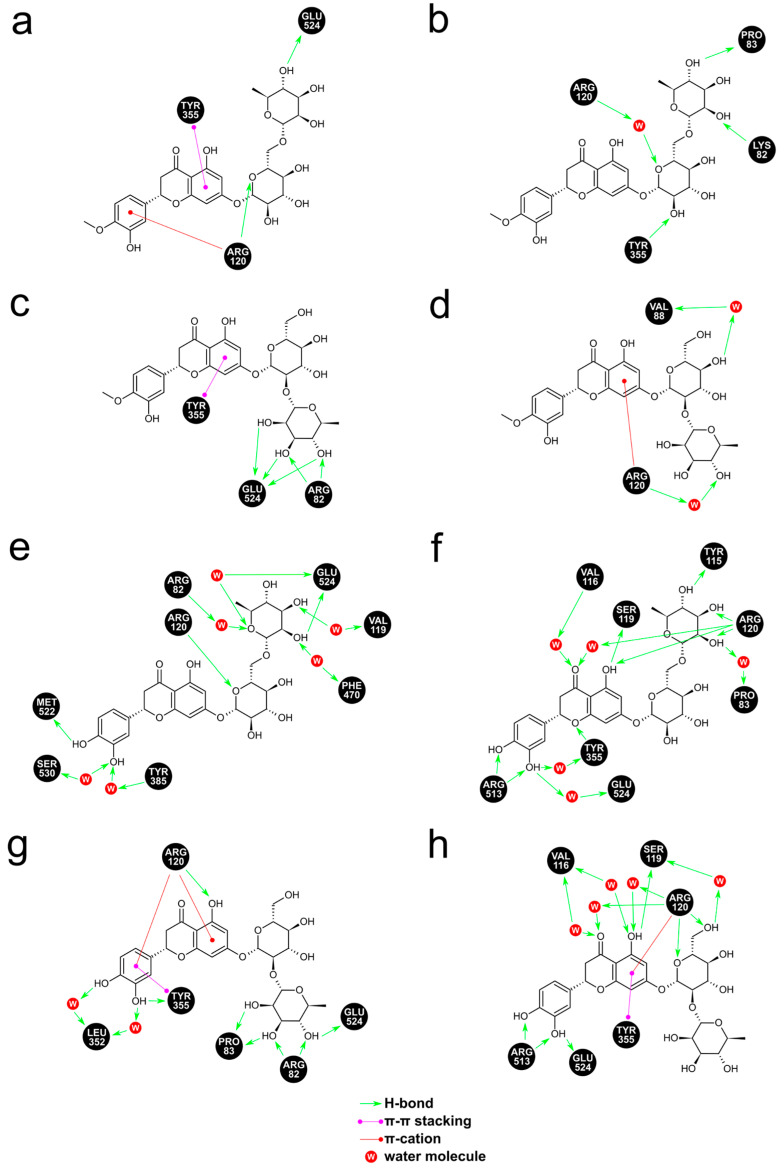
Protein/ligand interaction of (**a**) HED/COX-1; (**b**) HED/COX-2; (**c**) NHE/COX-1; (**d**) NHE/COX-2; (**e**) ERI/COX-1; (**f**) ERI/COX-2; (**g**) NER/COX-1, and (**h**) NER/COX-2.

Due to the similar binding modes, all compounds make analogous hydrophobic interactions with the COX-1 residues Val349, Leu359, Leu352, Leu357, Tyr355, Val119, Leu93, Leu115, Leu112, Pro83, Pro85, Met522, Ile523, Phe518, Trp387, Leu384, Ala527, Leu531, and Val116 (Figure 1a). The four phytocompounds showed a similar binding pattern to COX-2, with the ligands surrounded by the same, mainly hydrophobic, environment consisting of the aminoacidic residues Val88, Pro85, Pro83, Val116, Tyr115, Leu92, Ile112, Tyr355, Val523, Ile517, Phe518, Ala527, Leu531, Leu352, Leu359, and Val349 (Figure 1b). To better investigate the stability and the interactions of each ligand/protein complex predicted by the docking simulations, we challenged the best scoring docking poses by 48ns long molecular dynamics simulations. During the MD simulations, the ligands engaged in several ligand/protein hydrogen bonding interactions, both direct and water-mediated, and in π–π stacking and π–cation interactions. These interaction patterns are summarized in Table 3 and Figure 2.

During the MD simulations of the compounds bound to COX-1, HED (Figure 2a) makes hydrogen bonds with Glu524 and Arg120 by its disaccharide moiety. By its flavanone moiety, HED forms a π–cation interaction with Arg120 and π–π stacking with Tyr355. NHE (Figure 2c) makes hydrogen bonds between its disaccharide moiety and Glu524 and Arg83. The flavanone moiety of NHE forms π–π stacking with Tyr355. The disaccharide moiety of ERI (Figure 2e) makes hydrogen bonds with Arg120 and water-mediated hydrogen bonds with Glu524, Arg82, Val119, and Phe470. Moreover, the flavanone moiety of ERI makes a hydrogen bond with Met522 and water-mediated H-bonds with Ser530 and Tyr385. NER (Figure 2g) forms hydrogen bonds with Pro83, Arg82, and Glu524 through its disaccharide moiety. The flavanone moiety of NER forms a hydrogen bond with Arg120 and a water-mediated hydrogen bond with Leu352. Moreover, by its flavanone moiety, it forms π–cation interactions with Arg120 and π–π stacking with Tyr355.

During the MD simulations of the compounds bound to COX-2, HED (Figure 2b) makes hydrogen bonds with Tyr355, Lys82, and Pro83 and a water-mediated H-bond with Arg120 through its disaccharide moiety. NHE (Figure 2d) makes two water-mediated hydrogen bonds with Val88 and Arg120 through its disaccharide moiety, and its flavanone moiety forms a π–cation interaction with Arg120. ERI (Figure 2f) makes hydrogen bonds between its disaccharide moiety and Tyr115 and Arg120 and a water-mediated H-bond with Pro83. The flavanone moiety of ERI forms hydrogen bonds with Ser119, Arg120, Tyr355, and Arg513 and water-mediated H-bonds with Val116, Tyr355, Arg120, and Glu524. NER (Figure 2h) forms two hydrogen bonds with Arg120 and a water-mediated H-bond with Ser119 through its disaccharide moiety; moreover, its flavanone moiety makes hydrogen bonds with Glu524, Arg513, and Ser119 and water-mediated H-bonds with Arg120 and Val116. NER also makes a π–cation interaction with Arg120 and π–π stacking with Tyr355 through its flavanone moiety.

It is worth noting that HED and ERI, and NHE and NER, at position 7 of the flavanone share the same disaccharide moieties, i.e., rutinoside and neohesperidoside, respectively, while the couples HED/NHE and ERI/NER share the same aglycone, i.e., hesperetin and eriodictyol, respectively. Thus, from a structure–activity relationship point of view, the different activity profiles should be explained based on the methylation of the hydroxy group at the *para* position of the phenyl substituent. On the other hand, flavanones have been extensively studied and reported as potential NSAIDs [1,44]; in particular, eriodictyol has been reported as a selective COX-2 inhibitor [45].

MD trajectories were used to estimate the difference in protein/ligand interaction energy (see Section 2.4.) for each ligand between the two COX isoforms (ΔE_COX-2-COX-1_). The resulting values (Table 4) suggest a marked selectivity towards COX-2 of the eriodyctiol-based compounds, i.e., ERI and NER.

It is reported that the presence of Arg513 in COX-2 increases the polarity of the active site [46], whereas in the active site of COX-1, Arg513 is replaced by the smaller histidine residue that, in turn, is masked by the bulky Ile523 residue. 

**Figure 3:**
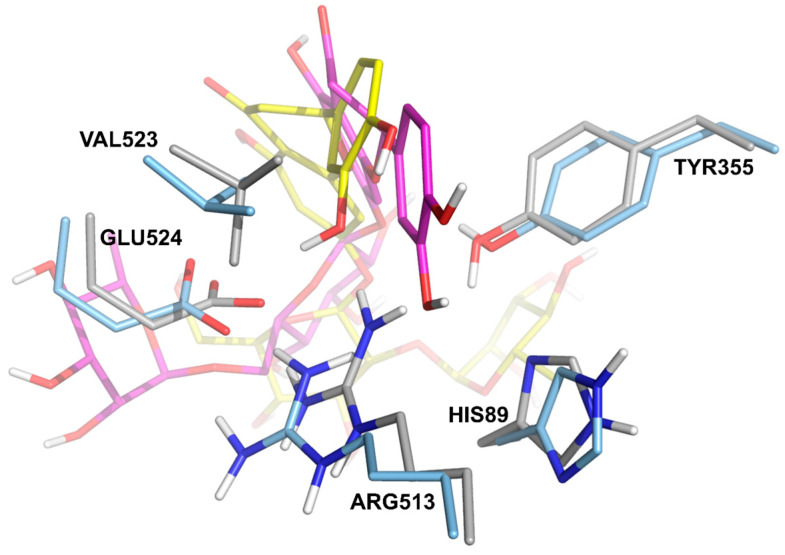
Molecular dynamics snapshots (at 6 ns simulation time) of ERI (magenta sticks) in complex with COX-2 (cyan sticks) and of NER (yellow sticks) in complex with COX-2 (gray sticks).

The I523V and the H513R mutations in COX-2 form a hydrophilic patch in the binding site that interacts optimally with the di-hydroxyphenyl moiety of the ERI and NER eriodyctiol (Figure 3), thus explaining their selectivity towards COX-2, whilst the more hydrophobic aglycone of HED and NHE does not fit very well for this interaction. Indeed, during the MD simulations of HED and NHE, their phenyl moieties point towards the less hydrophilic environment lined by Val349, Leu352, Tyr385, and Phe518. This region is conserved across the two COX isoforms, thus explaining the lack of selectivity shown by HED and NHE.

### 3.3. Anti-Inflammatory Activity

**Figure 4:**
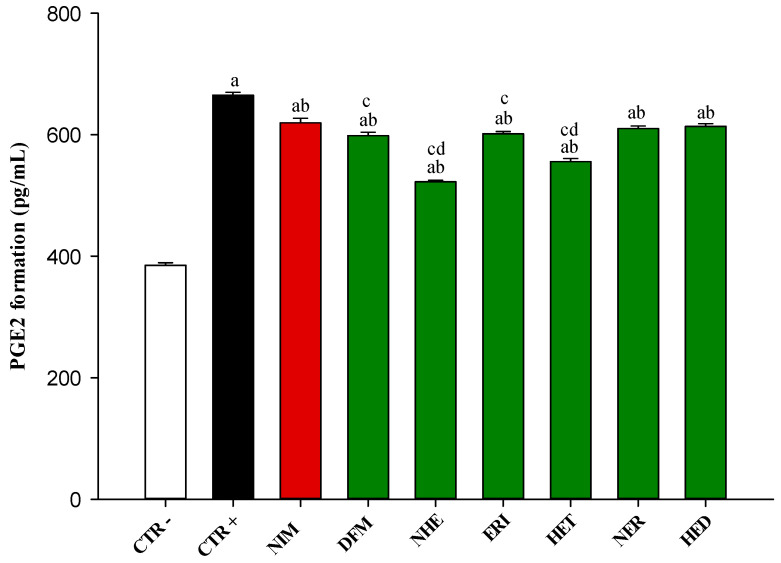
Prostaglandin (PGE2) release upon exposure of Caco-2 monolayers to 10 mM arachidonic acid (AA) after treatment with 25 ng/mL IL-1β. CTR−, negative control treated only with 10 mM AA; CTR+, positive control treated both with 25 ng/mL IL-1β and AA; NIM, nimesulide, used as reference standard; DFM, 10 µM digested flavanone mix; NHE, 10 µM neohesperidin; ERI, 10 µM eriocitrin; HET, 10 µM hesperetin; NER, 10 µM neoeriocitrin; HED, 10 µM hesperidin. ^a^ *p* < 0.001 vs. CTR−; ^b^ *p* < 0.001 vs. CTR+; ^c^ *p* < 0.05 vs. NIM; ^d^ *p* < 0.05 vs. DFM.

Given the marked inhibitory activity found in vitro in the preliminary enzymatic tests on human COX isoforms, also corroborated by molecular modeling studies, the ability of the five flavanones and of DFM to inhibit prostaglandin release after treatment with IL-1β and arachidonic acid was evaluated on a Caco-2 cell-based model. Nimesulide (10 µM) was used once again as a reference standard. The results, reported in Figure 4, show a statistically significant increase (*p* < 0.001 vs. CTR−) of PGE2 release following treatment with IL-1β (CTR+).

All treatments statistically significantly reduced PGE2 release with respect to CTR+. Interestingly three of the five flavanones, that is, NHE (*p* < 0.001), ERI (*p* < 0.05), and HET (*p* < 0.001), as well as DFM (*p* < 0.05) showed a significantly higher decrease than nimesulide, tested at the same concentration (10 µM). Furthermore, it seems that among the five tested flavanones, NHE and HET are primarily responsible for this activity.

### 3.4. Antioxidant Activity

An imbalance of the redox homeostasis with an overproduction of ROS is often associated with the inflammatory phenomenon. The ability of the tested flavanones and DFM to maintain this subtle balance by hindering the pro-inflammatory process induced by IL-1β was evaluated on the same cellular model by recording the changes in four key oxidative stress markers: carbonylated proteins, TBARS, ROS release, and GSH/GSSG ratio. Trolox was used as a reference standard due to its proven antioxidant activity.

**Figure 5:**
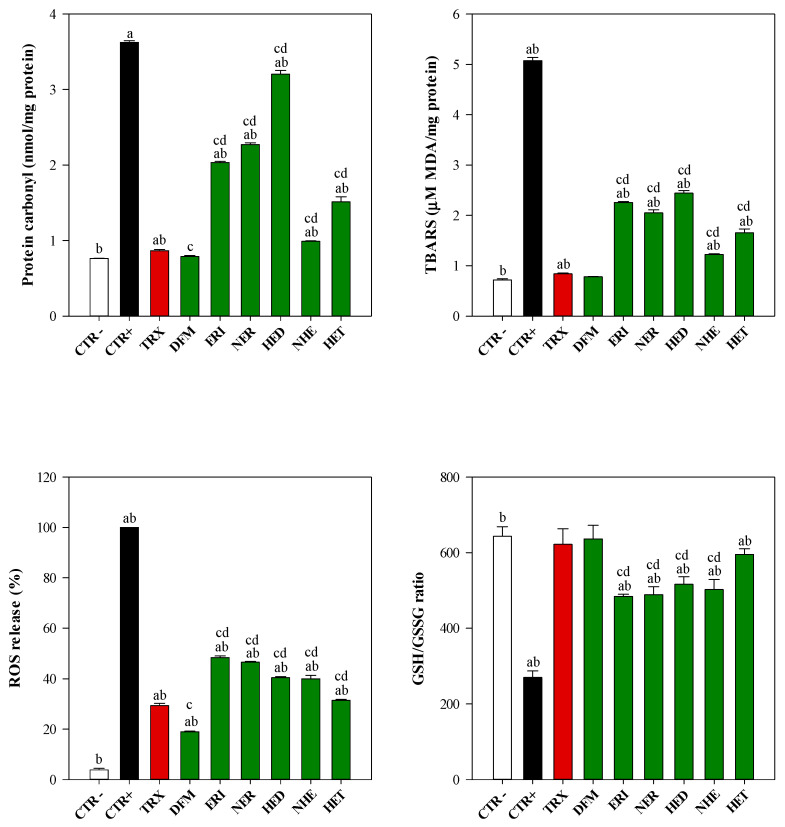
Exposure to 25 ng/mL IL-1β causes pro-oxidant response in Caco-2 monolayers. CTR−, negative control treated only with culture medium; CTR+, positive control treated with 25 ng/mL IL-1β; TRX, 10 µM Trolox, used as reference standard; DFM, 10 µM digested flavanone mix; NHE, 10 µM neohesperidin; ERI, 10 µM eriocitrin; HET, 10 µM hesperetin; NER, 10 µM neoeriocitrin; HED, 10 µM hesperidin. ^a^ *p* < 0.001 vs. CTR−; ^b^ *p* < 0.001 vs. CTR+; ^c^ *p* < 0.05 vs. TRX; ^d^ *p* < 0.05 vs. DFM.

As shown in Figure 5, IL-1β caused a significant increase in the protein carbonyl content, TBARS and ROS release, and a significant decrease in the GSH/GSSG ratio with respect to CTR−. DFM showed the strongest antioxidant activity in all four tests performed, showing, at the same concentration (10 µM), an activity comparable to trolox in terms of TBARS and GSH/GSSG ratio, or even higher (*p* < 0.05 vs. TRX) as in the case of carbonylated proteins and ROS release. According to previous results, a combination effect of the five tested flavanones is evident. Indeed, if tested alone at the same concentration (10 µM), they always show a significantly lower antioxidant activity (*p* < 0.05) than DFM. Interestingly, according to the first mentioned results on PGE2 release, the flavanones that seem to play a pivotal role in the DFM activity are the hesperidin glycosides NHE and HET (Figure 5).

### 3.5. Post-Quality Control Assays

No cytotoxicity (cell viability ≥ 98.27% ± 2.58) or alteration of the cell membrane permeability (TEER ≥ 800 Ω/cm^2^, LY Papp ≤ 1.08 × 10^−6^ cm/s and Pf ≤ 0.42%) were detected in the Caco-2 monolayers after antioxidant and anti-inflammatory assays, with these values remaining within the reference standard range [47,48].

### 3.6. Evaluation of the Synergistic Effects

**Figure 6:**
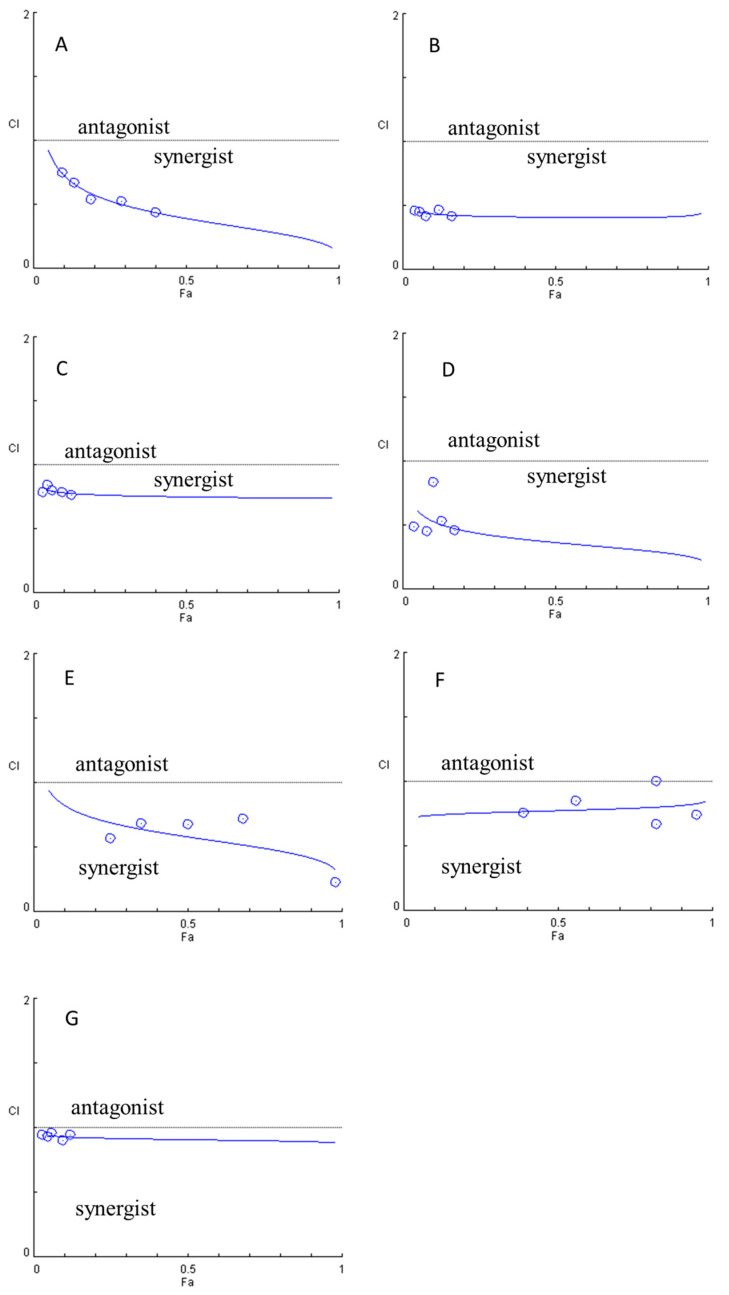
Screening of the synergistic effect of DFM (5–20 µM) at constant ratio of 2.0 by combining HED, NHE, HET, ERI, and NER, with each one at the following concentrations: 1.0–4.0 µM. The simulated lines were generated from CompuSyn by plotting CI versus Fa values. (**A**) ROS, (**B**) TBARS, (**C**) GSH/GSSG, (**D**) protein carbonyl, (**E**) COX-1, (**F**) COX-2, and (**G**) PGE2.

The potential synergistic effect of DFM was investigated using constant ratio experiments. For this purpose, all the antioxidant and anti-inflammatory assays carried out on the target concentration (10 µM) were repeated with two lower concentrations (5 and 7.5 µM) and one higher concentration (20 µM), to follow the DFM behavior at almost four concentrations. While the ratio of HED, NHE, HET, ERI, and NER was fixed at 2.0 for all experimental conditions, the concentration of the five flavanones in these analyses ranged from 1.0 to 4.0 µM. Figure 6 shows the DFM behavior in all the antioxidant (A–D) and anti-inflammatory (E–G) assays carried out.

The synergistic effect of the constant ratio of the tested concentrations of HED, NHE, HET, ERI, and NER was analyzed by CompuSyn software, which generates computerized simulation data from various concentrations and fractions of effect (Fa). The simulated data were simplified as a combination index plot (CI-Fa, Figure 6A–G). The synergistic effect of DFM was detected at every concentration point tested in each experimental condition (Figure 6A–G). Of note, the antioxidant and anti-inflammatory activity of DFM remained unchanged within the concentration range tested (5 to 20 µM) (Figure 6). The results indicate that at this ratio (2.0), HED, NHE, HET, ERI, and NER showed synergy on a broad range of effects showing IC values ≤ 0.90 with a confident limit of 90% for all tested experimental conditions. 

## 4. Discussion

The antioxidant power of flavonoids is now universally recognized and the number of studies available that support this activity, both in vitro and in vivo, is extremely high [1,2,49,50,51,52]. The effects of *Citrus* flavanones have been widely described in the literature and the health effects of these molecules are quite numerous, with various applications in the prevention and treatment of various chronic diseases, such as cardiovascular and metabolic disorders [1,35].

The antioxidant activity of these flavonoids is strongly related to the structure, functionalization, and spatial arrangement of the substituents, which neutralize ROS in various ways, transferring protons or electrons and quenching the radicals. Exposure to important levels of oxygen radicals is also related to the oxidation of GSH into GSSG, increasing lipid peroxidation that leads to unstable lipid peroxide derivatives from polyunsaturated fatty acids, and the generation of carbonyl groups on various amino acid residues such as lysine, arginine, proline, or threonine, leading to a cascade increase in oxidative stress [53,54,55]. For this reason, in addition to the scavenging capacity against ROS, it is important to evaluate some of the downstream effects of the antioxidant activity of flavanones, such as their ability to reduce carbonyl groups, TBARS, or, on the contrary, to increase the GSH/GSSG ratio, as demonstrated for the first time in the present study.

The detected antioxidant activity of these molecules can be ascribed to the number of hydroxyl groups (–OH) and their spatial arrangement, as in the case of eriocitrin, which has two –OH groups linked to the B ring in the –*ortho* position [56,57]. Indeed, several studies have highlighted how the reduction in the number of –OH groups is inversely proportional to the anti-radical action [58,59,60,61,62]. If the presence of –OH groups enhances the antioxidant activity, on the other side, a weakening factor is the glycosylation; for example, HED and NHE have a lower antioxidant efficacy than the aglycone HET [58,59]. The results obtained from the preliminary antioxidant screening conducted by our research group [20] showed a structure–activity relationship in line with what was previously assumed; in fact, eriocitrin and neoeriocitrin, glycosylated compounds of eriodyctiol, show a higher average antioxidant activity than HED, NHE, and their aglycon HET, probably due to the presence of a higher number of –OH, which, in HET and glycosylated derivatives, are replaced by methoxy groups (-OMe). Furthermore, HET proves to be, on average, more active than its two glycosides, confirming that the bound sugar portion reduces the antioxidant power [20].

From the preliminary screening, it can also be noted that HED, NHE, HET, ERI, and NER have, on average, a greater antioxidant action than other flavanones typical of the *Citrus* genus, such as diosmin and naringin [20]. Also in this case, the action is dependent on the structure; in fact, diosmin has a skeleton similar to HET, with an unsaturation in position 2 of the C ring, therefore having less hydrogen to donate in the antioxidant processes that use this mechanism of action. Methoxylation in position 4 of ring B also makes the molecule less active, as the bond dissociation energy is higher, given that the hydrogen bridge is only available on the starting phenol, whereas it is lost by reaction with the radical. Conversely, the adjacency between two –OH groups placed on the B ring reduces the dissociation energy, as both the phenol and the phenoxyl radical that are formed remain stable, as in the case of ERI and NER. Naringin, on the other hand, is on average less active, probably because, despite the structural similarity with the stronger molecules and the lack of bound sugars, it has a lower –OH number, which reduces its antioxidant power.

However, when passing from an in vitro cell-free model to an in vitro cell-based model, differences are often found and are associated with the solubility of the molecules in cellular media and their ability to interact with cell membranes. Many glycosides are, in fact, somewhat similar to those in vivo at the level of the intestinal lumen, better conveyed in the aqueous medium and thus reaching the intestinal barrier more easily, where they act mostly locally. However, it is now well known that some of them can also be easily absorbed by specific carriers, carrying out their activities at the systemic level [35,63,64,65,66]. From this point of view, it seems that di-glycosylated flavonoids have even greater affinity, as recently found for SGLT1 and GLUT-2 [66]. Since these compounds must exert their antioxidant and anti-inflammatory action locally, it is important to know what their real availability in the intestinal compartment after digestive processes is. Although several studies are available on the antioxidant and anti-inflammatory activity of some *Citrus* flavanones, such as HED, HET, and naringenin [67,68,69,70], the only study available to date in which a screening of the most representative flavanones of the *Citrus* genus was carried out with the same methods, and which therefore carried out a real comparison between the different molecules in the same experimental conditions, is that of Denaro et al. [20]. Among other factors, once the five most promising molecules (HED, HET, NHE, ERI, and NER) had been identified, the authors mixed them in an equimolar ratio, creating a mix of flavanones, called FM, with the aim of also evaluating a potential combination effect [20], as it is often observed for different *Citrus* extracts or juices [56,71].

They also simulated human gastro-duodenal digestion in vitro to mimic what happens in vivo and to evaluate the fate of these compounds once they reach the gastrointestinal tract, demonstrating that there is no change in or alterations to the five flavanones during digestion [20]. These results are consistent with what has already been observed in vitro and in vivo for some of the flavanones investigated, such as HED, NHE, and HET [72]. A possible explanation for this behavior lies in the peculiarity of the metabolism of the glycosylated compounds with rutinose and neohesperidose, which normally reach the distal part of the intestine intact, where they are hydrolyzed by the enzymes of the intestinal microbiota and finally absorbed [73].

Considering this, the results obtained from the previous study as well as those obtained in the present study are mainly attributable to the peculiar structure of the five selected flavanones: two rutinosides (HED and ERI), two neohesperidosides (NHE and NER), and one aglycone (HET).

Regarding the anti-inflammatory activity, there are several studies available that have investigated the ability of flavanones to modulate the inflammatory response [1,8,12]. In our first study [20], we demonstrated how DFM was able, at doses compatible with a dietary intake [1], to reduce the release of pro-inflammatory markers such as IL-6, IL-8, and NO after the IL-1β stimulation of Caco-2 cells [20].

The ability of flavanones to modulate inflammation depends on several factors, such as the antioxidant power and the inhibitory action of flavanones on key enzymes responsible for the activation and transduction of inflammatory stimuli. For example, HED is capable of inhibiting phosphodiesterase and mitogen-activated protein kinase (MAPKs) [74]. The latter have an important role in the intracellular signaling pathway during the inflammatory response, with it being strongly related to the nuclear factor kappa-light-chain-enhancer of activated B cells (NF-Κb) signaling pathway, which has a key role in modulating the gene expression of iNOS, COX-2, IL-6, and tumor necrosis factor (TNF)-α [74]. Furthermore, it has been demonstrated that flavanones downregulate NF-κB, although the differences in the experimental models used for this purpose affect the results obtained, which appear discordant and difficult to interpret [75].

Another important gap in the literature available to date is linked to the action of flavanones on COX, known for their involvement in IBD; in fact, more generally, the effects of flavonoids on COX expressed at the intestinal level are still little known. In a study carried out by López-Posadas et al. [75], the structure–activity relationships of some flavonoids, including HET, on the expression of COX-2 on LPS-stimulated IEC18 cells and the relative signaling pathways were investigated. The authors demonstrated that the expression of COX-2 was strictly related to the presence of free –OH groups, whereas it did not seem to be induced by the presence of methoxy groups (-OMe) [75]. Furthermore, the presence of -OH groups in position 4 of the B ring, or in position 5 of the A ring, reduces the phosphorylation of the nuclear factor of kappa light polypeptide gene enhancer in B-cells inhibitor (IkB) [75]. Considering this, it is possible to assume that the action of flavanones in the in vitro screening on COX enzymes is linked to the presence of free –OH; in fact, HET and its glycosylated derivatives have –OH in position 5 of ring A, and –OMe in position 4 of the B ring, whereas ERI and NER have two free –OHs on the B ring, in position 4 and 3. Moreover, the preliminary screening shows a difference in the selectivity of molecules; HET and its glycosides (HED and NHE) exhibit similar inhibitory activity on both COX-1 and COX-2, whereas ERI and NER show selectivity towards COX-2. These results have been confirmed by molecular modeling studies, which have allowed us to shed light on the different types of interactions that HED, NHE, NER, and ERI establish with the active site of COX-2, allowing us to hypothesize a rational use of these molecules as potent selective COX-2 inhibitors. These results agree with what has already been observed for eriodyctiol [45], the aglycone of ERI and NER. Eriodyctiol is, in fact, able to establish two hydrogen bonds with Tyr-371 and Ser-516 within the active site of COX-2, and only one hydrogen bond with Met-521 of COX-1, showing selective COX-2 activity [45]. However, the present study demonstrates, for the first time, that sugars further stabilize the structure, allowing the molecules to establish many more hydrogen bonds with the amino acid residues of the COX active site, with reference to COX-2. Beyond the behavior of each tested flavanone, what appears even more interesting is the behavior of DFM. It is, in fact, capable of inhibiting both COX isoforms, with a marked inhibitory activity on COX-2 (82%), almost superimposable with that of nimesulide (87.45%), a COX-2 selective non-steroidal anti-inflammatory drug. The activity of the five flavanones as well as of DFM was also investigated at the cellular level, evaluating the PGE2 release induced by IL-1β in the presence of arachidonic acid.

DFM reduces the PGE2 release comparable to nimesulide, whereas NHE and HET show a more marked action. These results do not exactly overlap those obtained in the in vitro enzymatic screening, for almost obvious reasons; in fact, it is impossible to compare two different experimental models if we consider that all processes which regulate the inflammatory signal take place at the cellular level and that, therefore, the action of these molecules depends not only on the direct interaction with COXs, but also on the ability to modulate the inflammatory pathways involved in their expression. For example, the transcriptional and translational regulation of NF-κB takes place regardless of the IkB phosphorylation processes circumventing the classical signaling pathway and using alternative pathways, and therefore, the notion that the flavanones can, based on their structure, modulate the inflammatory response in one way or another cannot be excluded [75]. Whatever this mechanism, it is unquestionable that flavanones modulate the expression of COXs and, even if the in vivo effects are difficult to predict, it is possible to assume that the expression of COXs, and of COX-2, as well as the production of prostaglandins, is downregulated by some flavanones in the presence of a potent oxidative stress [75].

Finally, it is important to underline that the most innovative aspect of this study is the experimental demonstration of a synergistic antioxidant and anti-inflammatory effect of the five selected flavanones when combined in a ratio of 2.0, with a CI value < 1 at 1.0–4.0 µM, which makes FM a potentially useful agent to counteract intestinal inflammation. 

Certainly, even if this study represents an upgrade compared to the previous one, as it has been useful to clarify one of the mechanisms underlying the anti-inflammatory activity of FM and has highlighted other targets on which this mix can act to reduce oxidative stress induced by inflammation, the next step is certainly to conduct an in vivo study in order to demonstrate the transability of what has been observed in vitro in cell-free and cell-based models on a suitable animal model of IBD.

## 5. Conclusions

This study demonstrates how the flavanones hesperidin, neohesperidin, hesperetin, eriocitrin, and eriocitrin exhibit strong antioxidant activity by reducing the release of reactive oxygen species, the formation of carbonylated proteins and lipid peroxides, and the oxidation of GSH to GSSG in Caco-2 cells. They are also able to exert strong anti-inflammatory activity by inhibiting COX enzymes, with a selectivity towards COX-2, as also demonstrated by molecular modeling studies, and consequently, the release of prostaglandins with an efficacy similar to the reference COX-2 selective drug nimesulide in Caco-2 cells. One of the limitations of this study is the use of a monoculture model, which does not allow the evaluation of the influence of the immune response to the inflammatory stimuli. Furthermore, other than demonstrating the effectiveness of the flavanone mix on intestinal inflammation, it would be interesting to evaluate the molecular pathways underlying the antioxidant and anti-inflammatory activity found.

However, the most interesting and innovative aspect of this study is the experimental demonstration that, when combined in a mixture at a constant ratio, these flavanones exert synergistic antioxidant and anti-inflammatory activity, which makes them certainly worthy of further in vivo studies for their potential use in the prevention and treatment of chronic inflammatory bowel disease.

## Figures and Tables

**Table 1:** Ramachandran plot results for COX-1_33-583_ and COX-2_33-583_ homology models. Results for COX-1 and COX-2 experimentally solved structures are shown as reference. Values were calculated with PROCHECK [27].

	COX-1_33-583_ Homology Model	COX-2_33-583_ Homology Model	Experimental COX-1 (PDB ID 6Y3C)	Experimental COX-2 (PDB ID 5IKR)
Residues in most favored regions	89.8%	89.7%	88.3%	90.0%
Residues in additional allowed regions	9.8%	10.1%	11.7%	9.8%
Residues in generously allowed regions	0.4%	0.0%	0.0%	0.1%
Residues in disallowed regions	0%	0.2%	0.0%	0.1%

**Table 2:** Inhibitory activity of the flavanones hesperidin (HED), neohesperidin (NHE), hesperetin (HET), neoeriocitrin (NER), eriocitrin (ERI), digested flavanone mix (DFM), and nimesulide (NIM) as a reference standard, on human COX-1 and COX-2 enzyme isoforms.

Sample	Inhibitory Activity (%)	
	COX-1	COX-2
HED	53.41 ± 0.21 ^a,c^	55.18 ± 0.28 ^a,b^
NHE	50.82 ± 0.28 ^a,d^	55.74 ± 0.42 ^a,b^
HET	55.51 ± 0.35 ^a,d^	58.30 ± 0.35 ^a,b^
NER	1.21 ± 0.02 ^a,b,d^	78.0 ± 0.14 ^a,b^
ERI	3.12 ± 0.01 ^a,b,d^	56.89 ± 0.08 ^a,b^
DFM	49.80 ± 0.18 ^a,d^	82.45 ± 0.15 ^a^
NIM	0.30 ± 0.01 ^b,d^	87.93 ± 0.12

^a^ *p* < 0.001 vs. NIM; ^b^ *p* < 0.001 vs. DFM; ^c^ *p* < 0.05 vs. COX-2; ^d^ *p* < 0.001 vs. COX-2.

**Table 3:** Interaction patterns of docking-predicted bound conformations of HED, ERI, NHE, and NER with COX-1 and COX-2, detected during MD simulations HB = H-bond; WHB = water-mediated H-bond; πC = π–cation and ππ = π–π stacking. * To facilitate comparison between the two COX isoforms, residue numbering of COX-1 is applied to both proteins.

Residue *	Residue Type		HED		ERI		NHE		NER	
	COX-1	COX-2	COX-1	COX-2	COX-1	COX-2	COX-1	COX-2	COX-1	COX-2
82	Arg	Lys			WHB		HB		HB	
83	Pro	Pro		HB		WHB			HB	
88	Thr	Val						WHB		
115	Leu	Tyr				HB				
116	Val	Val				WHB				WHB
119	Val	Ser			WHB	HB				WHB
120	Arg	Arg	HB, πC	WHB	HB	HB, WHB		WHB, πC	HB, πC	HB, WHB, πC
352	Leu	Leu							WHB	
355	Tyr	Tyr	ππ	HB		HB, WHB	ππ		ππ	ππ
385	Tyr	Tyr			WHB					
470	Phe	Phe			WHB					
513	His	Arg				HB				HB
522	Met	Met			HB					
524	Glu	Glu	HB		WHB	WHB	HB		HB	HB
530	Ser	Ser			WHB					

**Table 4:** Difference in interaction energies for each ligand between COX-2 and COX-1.

Ligand	ΔE_COX-2-COX-1_ ^a^
HED	−2.74
ERI	−38.83
NHE	3.22
NER	−16.34

^a^ kcal/mol.

## Data Availability

Not applicable.

## References

[1] Barreca D., Gattuso G., Bellocco E., Calderaro A., Trombetta D., Smeriglio A., Laganà G., Daglia M., Meneghini S., Nabavi S.M. (2017). Flavanones: *Citrus* phytochemical with health-promoting properties. Biofactors.

[2] Barreca D., Mandalari G., Calderaro A., Smeriglio A., Trombetta D., Felice M.R., Gattuso G. (2020). *Citrus* flavones: An update on sources, biological functions, and health promoting properties. Plants.

[3] Khan A., Ikram M., Hahm J.R., Kim M.O. (2020). Antioxidant and anti-inflammatory effects of citrus flavonoid hesperetin: Special focus on neurological disorders. Antioxidants.

[4] Bagetta D., Maruca A., Lupia A., Mesiti F., Catalano R., Romeo I., Moraca F., Ambrosio F.A., Costa G., Artese A. (2020). Mediterranean products as promising source of multi-target agents in the treatment of metabolic syndrome. Eur. J. Med. Chem..

[5] Rees A., Dodd G.F., Spencer J.P.E. (2018). The effects of flavonoids on cardiovascular health: A review of human intervention trials and implications for cerebrovascular function. Nutrients.

[6] Mahmoud A.M., Hernández Bautista R.J., Sandhu M.A., Hussein O.E. (2019). Beneficial effects of *Citrus* flavonoids on cardiovascular and metabolic health. Oxid. Med. Cell Longev..

[7] Musumeci L., Maugeri A., Cirmi S., Lombardo G.E., Russo C., Gangemi S., Calapai G., Navarra M. (2020). *Citrus* fruits and their flavonoids in inflammatory bowel disease: An overview. Nat. Prod. Res..

[8] Stevens Y., Rymenant E.V., Grootaert C., Camp J.V., Possemiers S., Masclee A., Jonkers D. (2019). The intestinal fate of *Citrus* flavanones and their effects on gastrointestinal health. Nutrients.

[9] Tanveer A., Akram K., Farooq U., Hayat Z., Shafi A. (2017). Management of diabetic complications through fruit flavonoids as a natural remedy. Crit. Rev. Food Sci. Nutr..

[10] Gandhi G.R., Vasconcelos A.B.S., Wu D.T., Li H.B., Antony P.J., Li H., Geng F., Gurgel R.Q., Narain N., Gan R.Y. (2020). Citrus flavonoids as promising phytochemicals targeting diabetes and related complications: A systematic review of in vitro and in vivo studies. Nutrients.

[11] Koolaji N., Shammugasamy B., Schindeler A., Dong Q., Dehghani F., Valtchev P. (2020). *Citrus* peel flavonoids as potential cancer prevention agents. Curr. Dev. Nutr..

[12] Smeriglio A., Marcoccia D., Denaro M., Trombetta D. (2022). Nutraceuticals in the treatment of inflammatory bowel disease: How the panorama has changed in the last decade?. Curr. Med. Chem..

[13] Salaritabar A., Darvishi B., Hadjiakhoondi F., Manayi A., Sureda A., Nabavi S.F., Fitzpatrick L.R., Nabavi S.M., Bishayee A. (2017). Therapeutic potential of flavonoids in inflammatory bowel disease: A comprehensive review. World J. Gastroenterol..

[14] Chang H., Lei L., Zhou Y., Ye F., Zhao G. (2018). Dietary flavonoids and the risk of colorectal cancer: An updated meta-analysis of epidemiological studies. Nutrients.

[15] Fusco R., Cirmi S., Gugliandolo E., Di Paola R., Cuzzocrea S., Navarra M. (2017). A flavonoid-rich extract of orange juice reduced oxidative stress in an experimental model of inflammatory bowel disease. J. Funct. Foods.

[16] Gholap P.A., Nirmal S.A., Pattan S.R., Pal S.C., Mandal S.C. (2012). Potential of *Moringa oleifera* root and *Citrus sinensis* fruit rind extracts in the treatment of ulcerative colitis in mice. Pharm. Biol..

[17] Khan R.A., Mallick N., Feroz Z. (2016). Anti-inflammatory effects of *Citrus sinensis* L., *Citrus paradisi* L. and their combinations. Pak. J. Pharm. Sci..

[18] He W., Li Y., Liu M., Yu H., Chen Q., Chen Y., Ruan J., Ding Z., Zhang Y., Wang T. (2018). *Citrus aurantium* L. and its flavonoids regulate TNBS-induced inflammatory bowel disease through anti-inflammation and suppressing isolated jejunum contraction. Int. J. Mol. Sci..

[19] Abe H., Ishioka M., Fujita Y., Umeno A., Yasunaga M., Sato A., Ohnishi S., Suzuki S., Ishida N., Shichiri M. (2018). Yuzu (*Citrus junos* Tanaka) peel attenuates dextran sulfate sodium-induced murine experimental colitis. J. Oleo. Sci..

[20] Denaro M., Smeriglio A., Trombetta D. (2021). Antioxidant and anti-inflammatory activity of *citrus* flavanones mix and its stability after in vitro simulated digestion. Antioxidants.

[21] Kamiloglu S., Capanoglu E., Bilen F.D., Gonzales G.B., Grootaert C., Van de Wiele T., Van Camp J. (2016). Bioaccessibility of polyphenols from plant-processing byproducts of black carrot (*Daucus carota* L.). J. Agric. Food Chem..

[22] The UniProt Consortium (2021). UniProt: The universal protein knowledgebase in 2021. Nucleic Acids Res..

[23] Xu S., Uddin M.J., Banerjee S., Duggan K., Musee J., Kiefer J.R., Ghebreselasie K., Rouzer C.A., Marnett L.J. (2019). Fluorescent indomethacin-dansyl conjugates utilize the membrane-binding domain of cyclooxygenase-2 to block the opening to the active site. J. Biol. Chem..

[24] Berman H.M., Westbrook J., Feng Z., Gilliland G., Bhat T.N., Weissig H., Shindyalov I.N., Bourne P.E. (2000). The protein data bank. Nucleic Acids Res..

[25] Thompson J.D., Higgins D.G., Gibson T.J. (1994). CLUSTAL W: Improving the sensitivity of progressive multiple sequence alignment through sequence weighting, position-specific gap penalties and weight matrix choice. Nucleic Acids Res..

[26] Waterhouse A., Bertoni M., Bienert S., Studer G., Tauriello G., Gumienny R., Heer F.T., de Beer T.A.P., Rempfer C., Bordoli L. (2018). SWISS-MODEL: Homology modelling of protein structures and complexes. Nucleic Acids Res..

[27] Laskowski R.A., MacArthur M.W., Moss D.S., Thornton J.M. (1993). PROCHECK: A program to check the stereochemical quality of protein structures. J. Appl. Cryst..

[28] Kim S., Chen J., Cheng T., Gindulyte A., He J., He S., Li Q., Shoemaker B.A., Thiessen P.A., Yu B. (2023). PubChem 2023 update. Nucleic Acids Res..

[29] Jones G., Willett P., Glen R.C., Leach A.R., Taylor R. (1997). Development and validation of a genetic algorithm for flexible docking. J. Mol. Biol..

[30] Bowers K.J., Chow E., Xu H., Dror R.O., Eastwood M.P., Gregersen B.A., Klepeis J.L., Kolossvary I., Moraes M.A., Sacerdoti F.D. Scalable Algorithms for Molecular Dynamics Simulations on Commodity Clusters. Proceedings of the ACM/IEEE Conference on Supercomputing (SC06).

[31] Alhindi T., Zhang Z., Ruelens P., Coenen H., Degroote H., Iraci N., Geuten K. (2017). Protein interaction evolution from promiscuity to specificity with reduced flexibility in an increasingly complex network. Sci. Rep..

[32] Jorgensen W.L., Maxwell D.S., Tirado-Rives J. (1996). Development and testing of the OPLS all-atom force field on conformational energetics and properties of organic liquids. J. Am. Chem. Soc..

[33] Jorgensen W.L., Chandrasekhar J., Madura J.D. (1983). Comparison of simple potential functions for simulating liquid water. J. Chem. Phys..

[34] Carbone D., Vestuto V., Ferraro M.R., Ciaglia T., Pecoraro C., Sommella E., Cascioferro S., Salviati E., Novi S., Tecce M.F. (2022). Metabolomics-assisted discovery of a new anticancer GLS-1 inhibitor chemotype from a nortopsentin-inspired library: From phenotype screening to target identification. Eur. J. Med. Chem..

[35] Denaro M., Smeriglio A., De Francesco C., Xiao J., Cornara L., Trombetta D. (2020). *In vitro* intestinal transport and anti-inflammatory properties of ideain across Caco-2 transwell model. Fitoterapia.

[36] Tesoriere L., Attanzio A., Allegra M., Gentile C., Livrea M.A. (2014). Indicaxanthin inhibits NADPH oxidase (NOX)-1 activation and NF-κB-dependent release of inflammatory mediators and prevents the increase of epithelial permeability in IL-1β-exposed Caco-2 cells. Br. J. Nutr..

[37] Smeriglio A., De Francesco C., Denaro M., Trombetta D. (2021). Prickly pear betalain-rich extracts as new promising strategy for intestinal inflammation: Plant complex vs. main isolated bioactive compounds. Front. Pharmacol..

[38] Nobili V., Alisi A., Mosca A., Crudele A., Zaffina S., Denaro M., Smeriglio A., Trombetta D. (2019). The antioxidant effects of hydroxytyrosol and vitamin e on pediatric nonalcoholic fatty liver disease, in a clinical trial: A new treatment?. Antioxid. Redox Signal..

[39] Kenzaoui B.H., Vilà M.R., Miquel J.M., Cengelli F., Juillerat-Jeanneret L. (2012). Evaluation of uptake and transport of cationic and anionic ultrasmall iron oxide nanoparticles by human colon cells. Int. J. Nanomed..

[40] Pengnam S., Plianwong S., Patrojanasophon P., Radchatawedchakoon W., Yingyongnarongkul B.E., Opanasopit P., Charoensuksai P. (2021). Synergistic effect of doxorubicin and siRNA-mediated silencing of Mcl-1 using cationic niosomes against 3D MCF-7 spheroids. Pharmaceutics.

[41] Xu S., Hermanson D.J., Banerjee S., Ghebreselasie K., Clayton G.M., Garavito R.M., Marnett L.J. (2014). Oxicams bind in a novel mode to the cyclooxygenase active site via a two-water-mediated H-bonding network. J. Biol. Chem..

[42] Kurumbail R.G., Stevens A.M., Gierse J.K., McDonald J.J., Stegeman R.A., Pak J.Y., Gildehaus D., Miyashiro J.M., Penning T.D., Seibert K. (1996). Structural basis for selective inhibition of cyclooxygenase-2 by anti-inflammatory agents. Nature.

[43] Blobaum A.L., Marnett L.J. (2007). Structural and functional basis of cyclooxygenase inhibition. J. Med. Chem..

[44] Rouzer C.A., Marnett L.J. (2020). Structural and chemical biology of the interaction of cyclooxygenase with substrates and non-steroidal anti-inflammatory drugs. Chem. Rev..

[45] Akinloye O.A., Metibemu D.S., Akinloye D.I., Onigbinde S.B., Olaosebikan I.A., Florence O., Damilola B., Bolarinwa O.A., Olubunmi O. (2019). Flavanones from *Sorghum bicolor* selectively inhibit COX-2: In-silico and in-vivo validation. Egypt J. Med. Hum. Genet..

[46] Sun H., Chow E.C., Liu S., Du Y., Pang K.S. (2008). The Caco-2 cell monolayer: Usefulness and limitations. Expert Opin. Drug Metab.Toxicol..

[47] Kamiloglu S., Capanoglu E., Grootaert C., Van Camp J. (2015). Anthocyanin absorption and metabolism by human intestinal Caco-2 cells–a review. Int. J. Mol. Sci..

[48] Smeriglio A., Barreca D., Bellocco E., Trombetta D. (2016). Chemistry, Pharmacology and Health Benefits of Anthocyanins. Phytother. Res..

[49] Smeriglio A., Barreca D., Bellocco E., Trombetta D. (2017). Proanthocyanidins and hydrolysable tannins: Occurrence, dietary intake and pharmacological effects. Br. J. Pharmacol..

[50] Smeriglio A., Calderaro A., Denaro M., Laganà G., Bellocco E. (2019). Effects of Isolated Isoflavones Intake on Health. Curr. Med. Chem..

[51] Gervasi T., Calderaro A., Barreca D., Tellone E., Trombetta D., Ficarra S., Smeriglio A., Mandalari G., Gattuso G. (2022). Biotechnological applications and health-promoting properties of flavonols: An updated view. Int. J. Mol. Sci..

[52] Kim H., Lee D.G. (2021). Naringin-generated ROS promotes mitochondria-mediated apoptosis in *Candida albicans*. IUBMB Life.

[53] Dalle-Donne I., Giustarini D., Colombo R., Rossi R., Milzani A. (2003). Protein carbonylation in human diseases. Trends Mol. Med..

[54] Ghani M.A., Barril C., Bedgood D.R., Prenzler P.D. (2017). Measurement of antioxidant activity with the thiobarbituric acid reactive substances assay. Food Chem..

[55] Smeriglio A., Cornara L., Denaro M., Barreca D., Burlando B., Xiao J., Trombetta D. (2019). Antioxidant and cytoprotective activities of an ancient Mediterranean *Citrus* (*Citrus lumia* Risso) albedo extract: Microscopic observations and polyphenol characterization. Food Chem..

[56] Diab K.A., Shafik R.E., Yasuda S. (2015). In vitro antioxidant and antiproliferative activities of novel orange peel extract and it’s fractions on leukemia HL-60 cells. Asian Pac. J. Cancer Prev..

[57] Bellocco E., Barreca D., Laganà G., Leuzzi U., Tellone E., Ficarra S., Kotyk A., Galtieri A. (2009). Influence of L-rhamnosyl-D-glucosyl derivatives on properties and biological interaction of flavonoids. Mol. Cell Biochem..

[58] Barreca D., Laganà G., Tellone E., Ficarra S., Leuzzi U., Galtieri A., Bellocco E. (2009). Influences of flavonoids on erythrocyte membrane and metabolic implication through anionic exchange modulation. J. Membr. Biol..

[59] Barreca D., Bellocco E., Caristi C., Leuzzi U., Gattuso G. (2011). Elucidation of the flavonoid and furocoumarin composition and radical-scavenging activity of green and ripe chinotto (*Citrus myrtifolia* Raf.) fruit tissues, leaves and seeds. Food Chem..

[60] Barreca D., Bisignano C., Ginestra G., Bisignano G., Bellocco E., Leuzzi U., Gattuso G. (2013). Polymethoxylated, C- and O-glycosyl flavonoids in tangelo (*Citrus reticulata* × *Citrus paradisi*) juice and their influence on antioxidant properties. Food Chem..

[61] Barreca D., Gattuso G., Laganà G., Leuzzi U., Bellocco E. (2016). C- and O-glycosyl flavonoids in Sanguinello and Tarocco blood orange (*Citrus sinensis* (L.) Osbeck) juice: Identification and influence on antioxidant properties and acetylcholinesterase activity. Food Chem..

[62] Lieder B., Hoi J.K., Holik A.K., Geissler K., Hans J., Friedl B., Liszt K., Krammer G.E., Ley J.P., Somoza V. (2017). The flavanone homoeriodictyol increases SGLT-1-mediated glucose uptake but decreases serotonin release in differentiated Caco-2 cells. PLoS ONE.

[63] Gauer J.S., Tumova S., Lippiat J.D., Kerimi A., Williamson G. (2018). Differential patterns of inhibition of the sugar transporters GLUT2, GLUT5 and GLUT7 by flavonoids. Biochem. Pharmacol..

[64] Kerimi A., Gauer J.S., Crabbe S., Cheah J.W., Lau J., Walsh R., Cancalon P.F., Williamson G. (2019). Effect of the flavonoid hesperidin on glucose and fructose transport, sucrase activity and glycaemic response to orange juice in a crossover trial on healthy volunteers. Br. J. Nutr..

[65] Zhang H., Hassan Y.I., Liu R., Mats L., Yang C., Liu C., Tsao R. (2020). Molecular mechanisms underlying the absorption of aglycone and glycosidic flavonoids in a Caco-2 BBe1 cell model. ACS Omega.

[66] Tejada S., Pinya S., Martorell M., Capó X., Tur J.A., Pons A., Sureda A. (2018). Potential anti-inflammatory effects of hesperidin from the genus citrus. Curr. Med. Chem..

[67] Salehi B., Fokou P.V.T., Sharifi-Rad M., Zucca P., Pezzani R., Martins N., Sharifi-Rad J. (2019). The therapeutic potential of naringenin: A review of clinical trials. Pharmaceuticals.

[68] Ferreira de Oliveira J.M.P., Santos C., Fernandes E. (2020). Therapeutic potential of hesperidin and its aglycone hesperetin: Cell cycle regulation and apoptosis induction in cancer models. Phytomedicine.

[69] Arafah A., Rehman M.U., Mir T.M., Wali A.F., Ali R., Qamar W., Khan R., Ahmad A., Aga S.S., Alqahtani S. (2020). Multi-therapeutic potential of naringenin (4′,5,7-trihydroxyflavonone): Experimental evidence and mechanisms. Plants.

[70] Smeriglio A., Denaro M., D’Angelo V., Germanò M.P., Trombetta D. (2020). Antioxidant, anti-inflammatory and anti-angiogenic properties of *Citrus lumia* juice. Front. Pharmacol..

[71] Giménez-Bastida J.A., Martínez-Florensa M., Espín J.C., Tomás-Barberán F.A., García-Conesa M.T. (2009). A *Citrus* extract containing flavanones represses plasminogen activator inhibitor-1 (PAI-1) expression and regulates multiple inflammatory, tissue repair, and fibrosis genes in human colon fibroblasts. J. Agric. Food Chem..

[72] Kobayashi S., Tanabe S., Sugiyama M., Konishi Y. (2008). Transepithelial transport of hesperetin and hesperidin in intestinal Caco-2 cell monolayers. Biochim. Biophys. Acta.

[73] Manthey J.A., Grohmann K. (1996). Concentrations of hesperidin and other orange peel flavonoids in *Citrus* processing byproducts. J. Agric. Food Chem..

[74] Parhiz H., Roohbakhsh A., Soltani F., Rezaee R., Iranshahi M. (2015). Antioxidant and anti-inflammatory properties of the *Citrus* flavonoids hesperidin and hesperetin: An updated review of their molecular mechanisms and experimental models. Phytother. Res..

[75] López-Posadas R., Ballester I., Mascaraque C., Suárez M.D., Zarzuelo A., Martínez-Augustin O., Sánchez de Medina F. (2010). Flavonoids exert distinct modulatory actions on cyclooxygenase 2 and NF-kappaB in an intestinal epithelial cell line (IEC18). Br. J. Pharmacol..

